# Disruption of Very-Long-Chain-Fatty Acid Synthesis Has an Impact on the Dynamics of Cellulose Synthase in *Arabidopsis thaliana*

**DOI:** 10.3390/plants9111599

**Published:** 2020-11-18

**Authors:** Xiaoyu Zhu, Frédérique Tellier, Ying Gu, Shundai Li

**Affiliations:** 1 Department of Biochemistry and Molecular Biology, Pennsylvania State University, University Park, PA 16802, USA; rainyzhu713@gmail.com; 2 Institut Jean-Pierre Bourgin, INRAE-AgroParisTech, 78000 Versailles, France; frederique.tellier@inrae.fr

**Keywords:** cellulose synthase complex, very-long-chain-fatty acid, Golgi, growth, *Arabidopsis*

## Abstract

In higher plants, cellulose is synthesized by membrane-spanning large protein complexes named cellulose synthase complexes (CSCs). In this study, the *Arabidopsis* PASTICCINO2 (PAS2) was identified as an interacting partner of cellulose synthases. PAS2 was previously characterized as the plant 3-hydroxy-acyl-CoA dehydratase, an ER membrane-localized dehydratase that is essential for very-long-chain-fatty acid (VLCFA) elongation. The *pas2-1* mutants show defective cell elongation and reduction in cellulose content in both etiolated hypocotyls and light-grown roots. Although disruption of VLCFA synthesis by a genetic alteration had a reduction in VLCFA in both etiolated hypocotyls and light-grown roots, it had a differential effect on cellulose content in the two systems, suggesting the threshold level of VLCFA for efficient cellulose synthesis may be different in the two biological systems. *pas2-1* had a reduction in both CSC delivery rate and CSC velocity at the PM in etiolated hypocotyls. Interestingly, Golgi but not post-Golgi endomembrane structures exhibited a severe defect in motility. Experiments using pharmacological perturbation of VLCFA content in etiolated hypocotyls strongly indicate a novel function of PAS2 in the regulation of CSC and Golgi motility. Through a combination of genetic, biochemical and cell biology studies, our study demonstrated that PAS2 as a multifunction protein has an important role in the regulation of cellulose biosynthesis in Arabidopsis hypocotyl.

## 1. Introduction

Cellulose is the major load-bearing component of the plant cell walls. In higher plants, cellulose is synthesized by plasma membrane-localized rosette-like structures called the cellulose synthase complexes (CSCs) [1,2]. In *Arabidopsis*, functional CSCs are composed of three distinct cellulose synthase isoforms (CESAs). CSCs specialized for cellulose synthesis in the primary cell wall is composed of CESA1-, CESA3-, CESA6- and CESA6-like proteins [3,4]. For cellulose synthesis in the secondary cell wall, CESA4, CESA7 and CESA8 are essential for secondary CSC formation [5,6,7]. Recent quantification of CESA stoichiometry suggests that CESA isoforms exist in 1:1:1 molecular ratio in both primary and secondary cell wall CSCs [8,9]. Live cell imaging has enabled the visualization of CSCs that move bi-directionally along trajectories defined by cortical microtubules [3,10,11,12]. Consistent with the hypothesis that CSCs synthesize cellulose only when they are localized at the plasma membrane [13,14], the diffraction-limited CSC particles remained immobile after delivery and moved laterally in the plane of plasma membrane at an average velocity of 350 nm/min [10]. The motility of CSCs is presumably driven by the polymerization or crystallization of cellulose microfibrils. Therefore, CSC velocity provides a valuable readout of the reaction rate in cellulose synthesis [15]. KORRIGAN1 was recently shown to be an integral part of both primary and secondary CSCs [16,17,18,19]. Other non-CESA proteins were shown to associate with primary CSCs, such as cellulose synthase interactive (CSI) proteins that bridge between CSCs and microtubules [20,21,22], and companion of cellulose synthase (CC) proteins that sustain microtubule and CSCs function during salt stress [23]. Organization of such a large protein complex requires tightly regulated assembly, trafficking and degradation/recycling of CSCs and their associated proteins from and to the plasma membrane. However, the investigation of CSCs in assembly, trafficking and degradation/recycling in plants is still in its infancy [24].

It is believed that CSCs are assembled in the Golgi. Early electron micrographs showed rosette- CSC structures at the trans-side of the Golgi and in Golgi-derived vesicles [25]; consistently, fluorescent protein-tagged CESAs were detected in Golgi bodies [3,10,11,12]. Golgi has been shown to play a role in guiding the insertion of CSCs to the plasma membrane through its interactions with the cytoskeleton [11]. In addition to plasma membrane and Golgi, CSCs localized to trans-Golgi network (TGN)/early endosome (EE) [11,12,16]. It is unclear whether TGN/EE-localized CSCs are involved in the secretory pathway and/or endocytic trafficking pathway due to the dual role of TGN/EE in plants. CSCs retrieval was observed in both interphase cells and in the developing cell plate [26,27]. CESAs were recently identified as cargo proteins of the classic adaptor protein 2 (AP2) complex of the clathrin-mediated endocytosis (CME) pathway [27]. TWD40-2, a putative member of the TPLATE complex, was also important for the endocytosis of CESAs [28]. CSCs also localized to intracellular compartments known as small CESA compartments (SmaCCs) or microtubule-associate cellulose synthase compartments (MASCs) [11,12]. SmaCCs/MASCs were less frequently observed in fast elongating cells in which CSC density is high, reflecting active cellulose synthesis in these cells, as compared to cells in which CSCs density is low, corresponding to slow growth and less active cellulose synthesis. Osmotic stress or treatment with cellulose synthase inhibitor can cause clearance of plasma membrane-localized CSCs and induce accumulation of SmaCCs/MASCs, suggesting that SmaCCs/MASCs might be involved in endocytic retrieval of CSCs upon stress [11,12]. A recent study showed that SmaCCs/MASCs accumulation was CME-dependent, supporting the importance of endocytosis in regulating cellulose synthesis [29]. This study also showed that SmaCCs/MASCs are critical for fast recovery of CSCs to the plasma membrane after abiotic stress is relieved, and the recovery process is dependent on CSCs-associated protein CSI1. However, SmaCCs/MASCs also associated with Golgi and TGN/EE were observed to associate with CSC delivery along microtubules [11,12]. The dual role of both TGN/EE and SmaCCs/MASCs complicates the dissection of CSC trafficking in plants. 

The *pas2-1* mutant was identified in an ethyl methane sulfonate (EMS) screen for altered response to cytokinin and defects in cell proliferation and differentiation [30]. PAS2 shares sequence similarity with the mammalian protein tyrosine phosphatase-like (PTPL) family, which contains a mutation in the conserved signature motif that rendered phosphatase inactivity [31,32,33,34]. Subsequent genetic and biochemical studies suggested that PAS2 interacts with cyclin dependent kinase A and negatively regulates G2/M cell cycle progression [35]. The yeast *phs1* mutant was also characterized as a cell cycle mutant defective in the G2/M phase [36]. PHS1 encodes a 3-hydroxy-acyl-CoA dehydratase, the third enzyme of the yeast Very-Long-Chain-Fatty-Acids (VLCFAs) elongase complex [37]. Similar to *phs1* mutants, *pas2-1* mutant was deficient in VLCFA production. Reciprocal complementation experiments suggest *Arabidopsis PAS2* and yeast *PHS1* are functionally equivalent [38]. Consistent with its function in VLCFA production, fluorescent protein tagged PAS2 associated with endoplasmic reticulum (ER) and co-localized CER10, another member of the VLCFA elongase complex [38]. Characterization of *pas2* confirmed that PAS2 encodes fatty acyl-CoA dehydratase and that it is a limiting step for VLCFA in plants. Given the importance of VLCFA as a structural component in sphingolipids, VLCFA is implicated in the secretory pathway for protein trafficking [38]. 

In this study, we provide evidence that PAS2 is important for cellulose biosynthesis. The PAS2 protein directly interacted with multiple CESAs, and in addition to its endoplasmic reticulum localization, it co-localized with CSCs in Golgi. Moreover, we found that a sufficient VLCFA level is required for efficient cellulose synthesis. We propose that PAS2 is involved in cellulose synthesis via its direct interactions with CESAs. Alternatively, PAS2 may affect cellulose production indirectly by mediating the VLCFA level.

## 2. Results

### 2.1. PAS2 Directly Interacts with CESA Proteins

**Figure 1:**
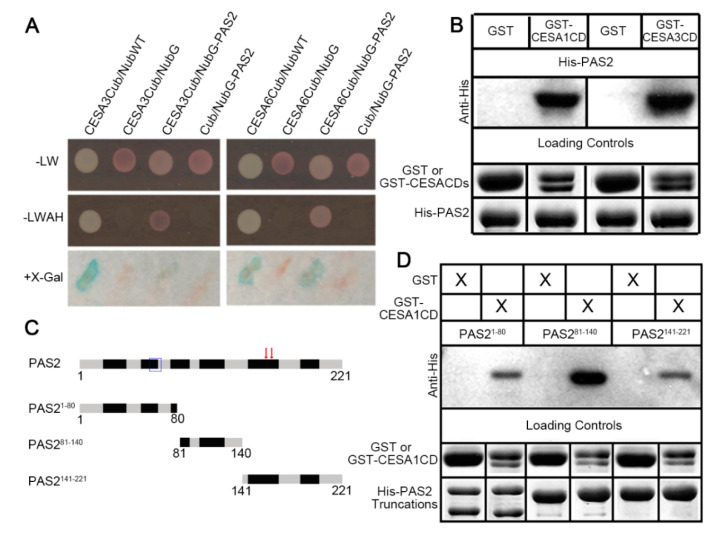
PAS2 interacts with multiple primary CESAs. (**A**) Split Ubiquitin yeast two hybrid analysis shows positive interactions between PAS2 and both CESA3 and CESA6. Interactions were selected on Leu (L), Trp (W), Ala (A) and His (H)-dropout medium. 5-Bromo-4-chloro-3-indolyl-β-D-galactopyranoside (X-Gal) was added for the detection of β-galactopyranoside activity. Cub vector alone serves as a negative control for NubG-PAS2. NubWT and NubG vectors serve as positive and negative controls, respectively, for CESA-Cub. (**B**) In vitro pull-down assay showing that PAS2 protein interacts with the central domains (CD) of CESA1 and CESA3. Both GST-CESA1CD and GST-CESA3CD co-precipitated with His-PAS2. Empty GST beads were used as negative controls that did not pull down His-PAS2. (**C**) Schematic graphs showing domain structure of PAS2. Black squares represent putative transmembrane domains. Blue box highlights the tyrosine phosphatase-like domain and red arrows represent the position of dehydratase’s catalytic residue Y156 and E163. (**D**) All three PAS2 truncations interact with the central domain of CESA1. GST-CESA1CD co-precipitated with all three truncated proteins tagged by His; Empty GST beads were used as negative controls.

To explore whether additional proteins may be involved in cellulose biosynthesis, we conducted a split-ubiquitin membrane-based yeast two-hybrid (SU-Y2H) screen to identify CESA interactive proteins. PAS2 was identified as a putative interaction partner of both CESA3 and CESA6 in the SU-Y2H screen. To confirm the interactions between CESA3/6 and PAS2, we subcloned full-length cDNA of PAS2 and tested its interaction with CESA3/6 using the SU-Y2H assay. In SU-Y2H assay, Wild-type Nub (NubWT) binds to the CESA3-Cub or CESA6-Cub, whereas a mutated form (NubG) does not, serving as positive and negative controls, respectively (Figure 1A). When PAS2 was fused to NubG (NubG-PAS2) and co-expressed with CESA3-Cub or CESA6-Cub in yeast, it fully reconstituted ubiquitin and activated both LacZ and HIS3/ADE2 (Figure 1A). 

To validate the interactions between PAS2 and CESAs, in vitro pull-down assays were performed. As we were unable to express full-length CESA proteins in *Escherichia coli*, we used central domains of CESAs (CESACD) in the pull-down assays [27]. Glutathione S-transferase (GST)-tagged central domains of CESA1 or CESA3 (GST-CESA1CD/GST-CESA3CD) were able to pull down Histidine (His)-tagged PAS2 (His-PAS2), while no detectable His-PAS2 was pulled down by purified GST (Figure 1B). We also attempted to specify the CESA-PAS2 interactive region by truncating the PAS2 protein into three fragments (Figure 1C). The first fragment contained the predicted tyrosine phosphatase-like domain, whereas the third fragment contained 3-hydroxyacyl-CoA dehydratase’s catalytic residues. Pull-down assays were performed between truncation proteins and GST-CESA1CD. Interestingly, all three truncations were pulled down by CESA1CD (Figure 1D). These data suggest that PAS2 directly associates with multiple primary cell wall CESAs.

### 2.2. Mutants of PAS2 Have Reduced Cellulose Contents and Reduced Motility of GFP-CESA3 

**Figure 2:**
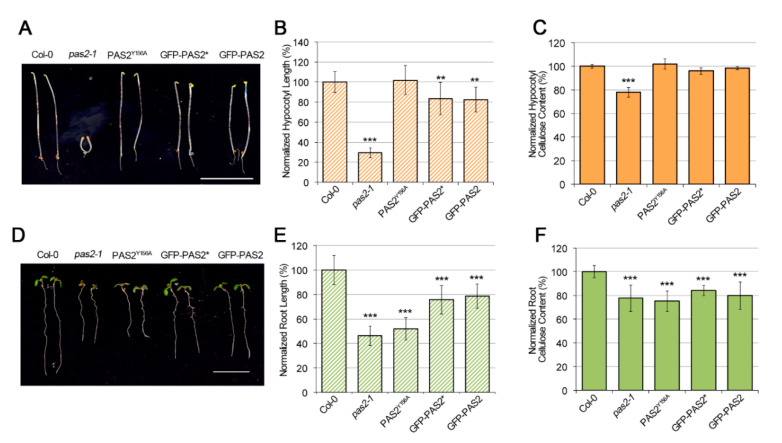
Phenotypic comparisons and cellulose content among wild-type (Col-0), *pas2-1*, GFP-PAS2^Y156A^, GFP-PAS2 and GFP-PAS2* seedlings. (**A**) Images of 4-day-old dark-grown seedlings. Bar = 1 cm. (**B**) Quantification of the length of 4-day-old etiolated hypocotyls. ** *p* < 0.001, *** *p* < 0.0001 (n ≈ 50 per transgenic line). Error bars represent SD. (**C**) Cellulose content of 4-day-old dark-grown seedlings. *** *p* < 0.0001. Error bars represent SD. (**D**) Images of 7-day-old light-grown seedlings. Bar = 1 cm. (**E**) Quantification of the root length. *** *p* < 0.0001 (n ≈ 50 per transgenic line). Error bars represent SD. (**F**) Cellulose content of 15-day-old dark-grown seedling roots. *** *p* < 0.0001. Error bars represent SD.

Mutations of *PAS2* led to strong developmental defects, consistent with its developmental regulation of transcripts in various tissues including seedling, root, leaf, stem and flower [30,33,34,39]. *pas2-*1 is an EMS mutant carrying an amino acid substitution of Gly to Ser [33,34]. *pas2-1* mutants could survive for up to 2 weeks on soil. *pas2-1* had shorter roots under a 16-h-light and 8-h-dark growth cycle (Figure 2D,E). The cell elongation defect was drastically manifested in dark-grown seedlings as *pas2-1* mutant has extremely short etiolated hypocotyls (Figure 2A,B), a feature that is often associated with cellulose deficiency of the primary cell wall [26,29,40]. To investigate whether the reduced cell elongation is associated with reduced cellulose production, the crystalline cellulose content in etiolated seedlings was measured in both dark-grown seedlings and light-grown roots [41]. *pas2-1* mutants had a 22.13% and 22.30% reduction in crystalline cellulose content in dark-grown seedlings and light-grown roots, respectively (Figure 2C,F).

**Figure 3:**
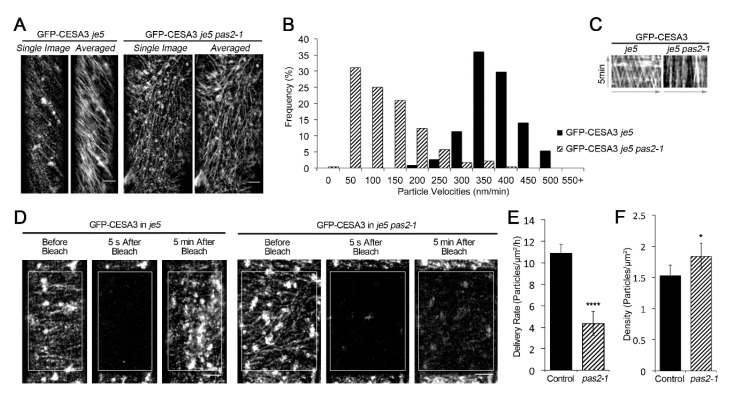
The *pas2-1* mutant has altered cellulose synthase dynamics. (**A**) Single-frame and time-averaged images from a 5-min time series (see “Materials and Methods”) of epidermal cell in etiolated seedlings show the plasma membrane-localized GFP-CESA3 particles in the *je5* and *je5 pas2-1* mutant background. Bar = 5 μM. (**B**) A histogram showing the distribution of GFP-CESA3 particle velocities. The mean velocity of GFP-CESA3 is 376.36 ± 55.35 nm/min (*n* = 114, 9 cells) in *je5* and 123.35 ± 79.89 nm/min (*n* = 228, 23 cells) in *je5 pas2-1*, respectively. (**C**) Kymographs displaying CESA3 particle movement from a single track in the averaged images. (**D**) Representative images displaying the plasma membrane CSCs before, 5 s after and 5 min after photo bleaching. FRAP was performed in the control (GFP-CESA3 in *je5*) etiolated seedlings and in *pas2-1* mutants (GFP-CESA3 in *je5 pas2-1*). White boxes mark the bleached area. Bar = 5 μM. (**E**) Quantifications of the delivery rate of CSCs from the FRAP assay described in D. **** *p* < 0.0001 (*n* = 26 ROIs from 5 seedlings for *je5*; *n* = 31 ROIs from 7 etiolated seedlings for *je5 pas2-1*). (**F**) A graph of the average density of plasma membrane-localized GFP-CESA3 particles. * *p* < 0.01 (*n* = 8 cells from 5 seedlings for *je5* and 17 cells from 9 seedlings for *je5 pas2-1*). Error bars represent SD.

To investigate whether the cellulose reduction in *pas2-1* reflects its defect in CSC dynamics or distribution, we introduced GFP-CESA3, a marker for CSC [3] in *pas2-1*. Consistent with the observed reduction in cellulose content, the motility of GFP-CESA3 was reduced in *pas2-1*. While the average velocity of CSCs is about 350 nm/min in wild type plants [10,20], the motility was much lower in *pas2-1* mutants (Figure 3A–C, Supplemental Video S1). Quantification of particle velocities showed that the average velocity of CSCs of *pas2-1* was reduced by 2/3 of that of wild-type plants (376.36 ± 55.35 nm/min for WT and 123.35 ± 79.89 nm/min for *pas2-1*, respectively). Consistently, the kymograph of each particle appeared as nearly straight parallel lines in *pas2-1* mutants instead of crossed oblique lines in WT (Figure 3C), indicating a very low velocity. 

Cellulose deficiency can also be reflected by defect in the delivery of CSCs to the PM. To investigate the delivery efficiency of CSCs, we performed a fluorescent recovery after photobleaching (FRAP) assay, in which the de novo delivery of CSC particles was monitored following photobleaching (Figure 3D). In the control line (GFP-CESA3 in *je5* background), the CESA delivery rate is 10.88 ± 0.88 particles/μm^2^/h; while in *pas2-1* mutants, the delivery rate was reduced drastically (4.35 ± 1.11 particles/μm^2^/h) (Figure 3E and Supplemental Video S2). We also evaluated the distribution of CSCs at the PM by analyzing the PM-localized GFP-CESA3 in *pas2-1* [27,28]. The particle density of PM-localized CESA in *pas2-1 je5* background was 1.84 ± 0.21 particles/μm^2^, slightly higher than that in the *je5* background (1.53 ± 0.17 particles/μm^2^) (Figure 3F). These observations suggest that the cellulose deficiency in *pas2-1* mutant is resulted from combined defects in both delivery and motility of CSCs. 

### 2.3. PAS2 Is Required for Proper Golgi Dynamics

**Figure 4:**
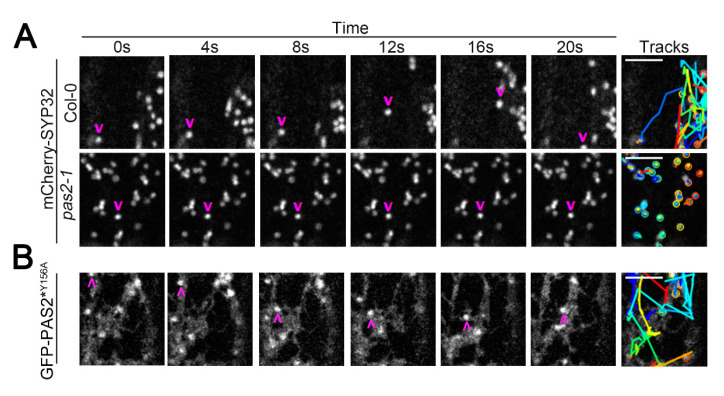
The *pas2-1* mutant specifically disrupts the Golgi motility, which was rescued by *pPAS2::*GFP-*PAS2^Y156A^*. (**A**) Analysis of Golgi motility in control (Col-0) and *pas2-1* seedlings. (**B**) Analysis of Golgi motility in *pas2-1* seedlings transformed with *pPAS2::*GFP-*PAS2^Y156A^*. A representative image and tracking analysis were shown (*n* = 30 per genotype). A series of single-frame images was taken to show the locations of mCherry-SYP32 and GFP-PAS2^Y156A^ labeled particles at multiple time points. Arrows indicate the location of one representative particle. Tracks indicate the trajectories of all particles (marked by circles) within 20 s. Different colors represent for different particles been tracked. Bar = 5 μM.

We noticed a defect in Golgi motility when we performed the FRAP experiment to examine the delivery rate of CESA in *pas2-1*. To further investigate the intracellular trafficking in the *pas2-1* mutants, we introduced a subset of organelle markers into *pas2-1*. *pas2-1* showed no obvious defect in particle morphology or dynamics for many organelle markers including the mCherry-SYP61 labeled TGN/EE, the mCherry-RabC1 labeled post-Golgi/endosome, the mCherry-RabA1g labeled endosomal/recycling endosome and the mCherry-RabG3f labeled endosome/vacuole (Figure S1). However, *pas2-1* drastically reduced the dynamics of mCherry-SYP32-labeled Golgi. Although mCherry-SYP32 had no morphological difference in the *pas2-1* mutants compared to wild type, instead of streaming bi-directionally along the longitudinal axis of the cell as in wild-type plants, mCherry-SYP32 in *pas2-1* mutants were barely motile and showed a wobbling motion (Figure 4A, Supplemental Video S3). 

### 2.4. Disruption of PAS2′s Dehydratase Activity Had Different Effects in Etiolated Hypocotyls and Light-Grown Roots

**Figure 5:**
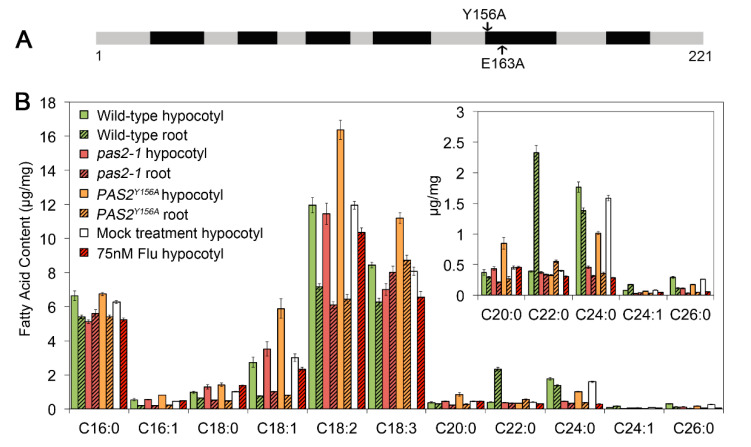
Both disruption of PAS2′s dehydratase activity and pharmacological inhibition lead to VLCFA depletion in hypocotyls and roots. (**A**) A schematic graph showing the position of two point mutations *PAS2^Y156A^* (left) and *PAS2^E163A^* (right), indicated by arrows. (**B**) Fatty acid methyl esters (FAMES) analysis of hypocotyls of 4-day-old etiolated seedlings and roots of 15-day-old light-grown seedlings. The *pPAS2::*GFP-*PAS2^Y156A^* plants showed differential extend of VLCFA decrease in hypocotyls and in roots; and 75nM Flu treatment mimicked the VLCFA pool in the *pas2-1* mutants, characterized by reduction in VLCFA content and increased short chain fatty acid portion. Moreover, the Flu treatment caused more severe depletion of VLCFA in the hypocotyls, comparing to *pPAS2::*GFP-*PAS2^Y156A^* seedlings.

Previous studies have shown that PAS2 is both structurally and functionally equivalent to the yeast PHS1, the 3-hydroxyacyl-CoA dehydratase that catalyzes the third reaction of the cyclic elongation of VLCFAs [38,42]. *pas2-1* mutants are deficient in the production of VLCFA, characterized by accumulation of 3-hydroxy-acyl-CoA intermediates [38]. As proper deposition of VLCFAs is critical for the regulation of membrane structure and dynamics in all eukaryotes [43], the activity of membrane-localized proteins such as CSCs might be influenced by the integrity of membranes. One possible scenario is that the cellulose synthesis defect (reduced cellulose content, CSC motility and delivery) in *PAS2* mutants is indirectly influenced by abnormal VLCFA production. To evaluate the effect of VLCFA defect on cellulose synthesis, we generated point mutations on the PAS2 protein to specifically disrupt its 3-hydroxyacyl-CoA dehydratase activity. It has been shown that for yeast PHS1, two amino acid residues, Tyr-156 and Glu-163, are indispensable for its dehydratase activity [37]. Sequence alignment showed that both of these two residues are highly conserved among eukaryotic organisms (Figure S2). Site-directed mutagenesis of cDNA sequence of *PAS2* was carried out to generate a construct carrying *GFP-PAS2^Y156A^* or *GFP-PAS2^E163A^* under the control of 1.5-kb *PAS2* native promoter (Figure 5A). Because *pas2-1* plants are seedling lethal, mutant constructs were transformed into plants of *pas2-1* heterozygous background, respectively. *pas2-1* homozygous plants containing *pPAS2::GFP-PAS2^Y156A^* or *pPAS2::GFP-PAS2^E163A^* transgene were identified in the subsequent generation. We used hygromycin to select around 50 T1 lines carrying either *pPAS2::GFP-PAS2^E163A^* or *pPAS2::GFP-PAS2^Y156A^.* We were able to identify *pas2-1* homozygous plants containing *pPAS2::GFP-PAS2^E163A^* or *pPAS2::GFP-PAS2^Y156A^* transgene. However, *pPAS2::GFP-PAS2^E163A^ pas2-1* plants yield only a few seeds and they failed to germinate. We were able to identify several *pPAS2::GFP-PAS2^Y156A^ pas2-1* and plants were fertile. Therefore, we focused our study on *pPAS2::GFP-PAS2^Y156A^ pas2-1* homozygous line. 

In contrast to the findings that PHS1^Y149A^ failed to complement lethality in yeast, GFP-PAS2^Y156A^ rescued the *pas2-1* seedling lethality phenotype and plants were fertile. Fatty acids methyl ester (FAMEs) assays showed that similar to *pas2-1*, *GFP-PAS2^Y156A^* had lower levels of long chain fatty acid in both light-grown roots and dark-grown hypocotyls, validating the essential function of Tyr156 for PAS2 dehydratase activity (Figure 5B). Both *pas2-1* and *GFP-PAS2^Y156A^* plants had a higher level of short chain fatty acid (C18:2 and C18:3) and lower levels of long chain fatty acid (C22:0, 24:0, 24:1 and 26:0). However, multiple VLCFA groups (C22:0, 24:0, 24:1 and 26:0) showed a more significant decrease in root than in hypocotyls. Interestingly, the VLCFA depletion led to growth defect in the roots but not in dark-grown hypocotyls. The *GFP-PAS2^Y156A^* line had comparable length of etiolated hypocotyl and crystalline cellulose content to that of wild type, but the elongation and cellulose content in light grown roots were both decreased (Figure 2). These data indicate that the dark-grown hypocotyls and light-grown roots have different regulation of VLCFA production. Disruption of PAS2′s dehydratase activity led to more pronounced reduction of VLCFA pool in roots than that in hypocotyls. Consistent with the preferential amount of VLCFA in two different systems, plant growth and cellulose content were more tightly correlated in roots as compared with that of hypocotyls (Figure 2). 

### 2.5. VLCFA Reduction by Flufenacet in Wild Type Hypocotyls to a Level Comparable to That of pas2-1 Mutants Did Not Cause Cellulose Synthesis Defect 

**Figure 6:**
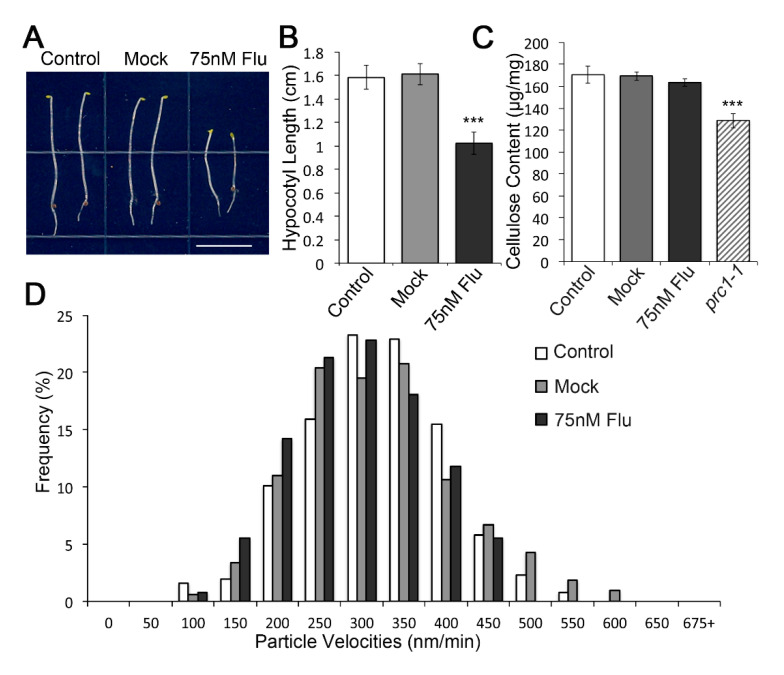
General VLCFA synthesis defect does not influence the functionality of the cellulose synthesis machinery. (**A**) Application of 75 nM Flu reduced the hypocotyl length of etiolated seedlings. 4-day-old dark-grown wild type (Col-0) seedlings grown on ½ MS medium (Control) or ½ MS medium containing 75 nM Flu or comparable amount of DMF (Mock). (**B**) Quantification of hypocotyl length of seedlings from the drug treatment analysis. *** *p* < 0.0001 (*n* ≈ 50 per experimental group). (**C**) Crystalline cellulose production was not affected following Flu treatment. Cellulose content of 3-day-old seedlings grown on ½ MS medium, ½ MS medium containing 75 nM Flu or comparable amount of DMF. Error bars represent SD. *** *p* < 0.0001. (**D**) A histogram showing the distribution of GFP-CESA3 particle velocities. The mean velocity of plasma membrane GFP-CESA3 for control, Mock and Flu groups were 319.59 ± 83.93 nm/min (*n* = 258, 14 cells from 6 seedlings), 318.21 ± 92.41 nm/min (*n* = 328, 16 cells from 7 seedlings) and 295.89 ± 79.00 (*n* = 127, 8 cells from 5 seedlings), respectively.

Given that the VLCFA depletion (C24:0) in *pas2-1* was more pronounced compared with that in *PAS2^Y156A^* in hypocotyls, it may partially explain the phenotype difference of dark-grown hypocotyls between *pas2-1* and *PAS2^Y156A^*. To test whether a cellulose synthesis defect observed in *pas2-1* mutant hypocotyls is due to the reduction in VLCFA content, we treated wild-type plants with the VLCFA elongase inhibitor Flufenacet [44,45]. Consistent with previous reports, for seedlings treated with 75 nM Flufenacet, the VLCFA was reduced to the level that is comparable to the hypocotyls of *pas2-1* mutants, while the mock treatment with DMF did not influence the VLCFA production (Figure 6A). Upon 75 nM Flufenacet treatment, the hypocotyl length of dark-grown seedlings was reduced to 63.39% of the mock treated seedlings (Figure 6A,B), while surprisingly, neither the crystalline cellulose content (163.45 ± 3.45 μg/mg compared to 169.22 ± 3.74 μg/mg in mock treatment) nor the plasma membrane CSC motility (295.89 ± 79.00 nm/min compared to 318.21 ± 92.41 nm/min in mock treatment) was influenced after Flufenacet treatment (Figure 6C,D). 

To sum up, although reduction in cellulose deposition in the roots suggested a role of VLCFA in cellulose synthesis, inhibition of VLCFA synthesis does not solely attribute to the cellulose deficiency in *pas2-1* mutants, at least in the etiolated hypocotyls.

### 2.6. Investigation of PAS2′s Subcellular Localization in Arabidopsis Hypocotyls

**Figure 7:**
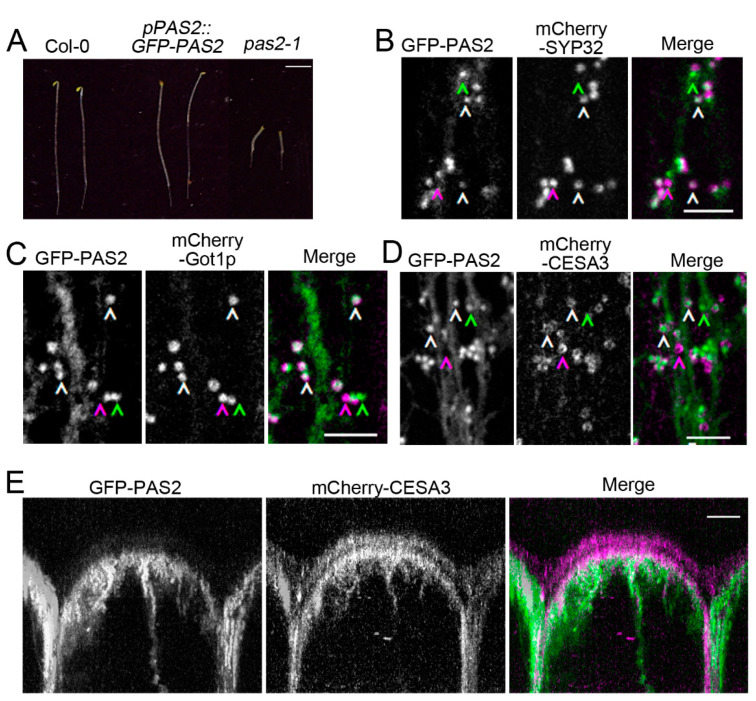
We observed that PAS2 localizes in Golgi and associates with CSCs in Golgi in our GFP-PAS2 transgenic line. (**A**) Image of 4-day-old dark-grown seedlings of wild type (Col-0), *pas2-1* and *pPAS2::GFP-PAS2* in *pas2-1* mutant background; *pPAS2::GFP-PAS2* was able to complement the *pas2-1* mutant phenotype. Bar = 0.5 cm. (**B**,**C**) Representative single-frame images show colocalization between GFP-PAS2 and two Golgi markers, mCherry-SYP32 and mCherry-Got1p, in the epidermal cells of etiolated hypocotyls. Manual particle selection was used to enhance the detection of colocalized particles. Green, magenta and white arrowheads denote GFP-, mCherry- and GFP-/mCherry- overlapped foci, respectively. Bars = 5 μm. (**D**) PAS2 associates with CSCs in Golgi. Representative single-frame images show colocalization between GFP-PAS2 and Golgi-localized mCherry-CESA3. Green, magenta and white arrowheads denote GFP-PAS2, mCherry-CESA3 and GFP-PAS2/mCherry-CESA3 overlapped foci, respectively. Bar = 5 μm. (**E**) PAS2 does not co-localize with the plasma membrane localized CSCs. A cross section of an epidermal cell from the etiolated *Arabidopsis* hypocotyl is displayed. mCherry-CESA3 signal localizes both at the plasma membrane and in intracellular space, representing plasma membrane-localized and intracellular CSCs; while GFP-PAS2 signal is absent from the mCherry-CESA3 decorated plasma membrane. Bar = 5 μm.

It has been reported that PAS2 localized exclusively to ER in the epidermal cells of tobacco leaves [38]. The subcellular localization is consistent with the function of PAS2 as an ER-resident VLCFA elongase. To examine the subcellular localization of PAS2 in *Arabidopsis*, we generated a construct containing green fluorescent protein, full-length cDNA of PAS2 and an approximately 1.5-kb native PAS2 promoter (*pPAS2::GFP-PAS2*). *pPAS2::GFP-PAS2* was transformed in *pas2-1* mutants. In the hypocotyl cells of 2.5-day dark-grown *Arabidopsis*, GFP-PAS2 localized to ER, which was consistent with previous observations, but also to other intracellular compartments with a shape and dynamic that is reminiscent of Golgi apparatus (Supplemental Video S4). Distinct fluorescent signals were detected in clusters of bright round-shaped structures that move bi-directionally along endoplasmic ER strands. To examine the identity of PAS2-labeled intracellular compartments, we crossed GFP-PAS2 to a subset of mCherry-labeled organelle marker lines [46]. Over 90% of PAS2 signals overlapped with Golgi markers (93.35% ± 4.90% colocalized with mCherry-SYP32 and 94.37% ± 3.13% colocalized with mCherry-Got1p) (Figure 7B,C). In most cases, PAS2 localized to the center of donut-shaped Golgi stacks labelled by mCherry-SYP32 and mCherry-Got1p. No overlap was observed between GFP-PAS2 and mCherry-VTI12, a putative early endosome/trans-Golgi network marker (Figure S4A). GFP-PAS2 neither colocalized with the endosome/recycling endosome marker mCherry-RabA1g nor the late endosome/vacuole marker mCherry-RabG3f (Figure S4B,C). These observations suggest that PAS2 localized both in ER and Golgi in *Arabidopsis* dark-grown hypocotyls.

Trafficking of CESA occurs mainly between plasma membrane, Golgi apparatus, the trans-Golgi network and SmaCCs/MASCs [47]. To investigate the relationship between CSCs and PAS2 in planta, a transgenic line was generated by crossing between GFP-PAS2 and mCherry-CESA3 [17]. mCherry-CESA3 localized to globular intracellular structures that have been previously identified as Golgi apparatus and to punctate particles at the PM that exhibit bi-directional motility. Two-channel confocal imaging revealed that GFP-PAS2 co-localizes with mCherry-CESA3 in cortical Golgi apparatus (Figure 7D). Most often, CESA surrounded PAS2 in the Golgi apparatus, and they circulated through the cytoplasm in a nonlinear pattern (Figure 7D, Supplemental Video S5). GFP-PAS2 signals were only detected in the cortex, whereas mCherry-CESA3 localized both PM and cortical intracellular organelles (Figure 7E). The distribution of mCherry-CESA3 to SmaCCs/MASCs was induced by 100 nM isoxaben, whereas GFP-PAS2 did not co-localize with mCherry-CESA3-associated SmaCCS/MASCs (Figure S4D and Supplementary Video S6). We also found that the GFP-PAS2^Y156A^ had normal localization to ER and Golgi (Figure S3) and displayed normal dynamics compared to that of GFP-PAS2 (Figure 2B, Supplemental Video S7). This indicates that disruption of PAS2′s dehydratase activity does not interfere with its localization in Golgi. Moreover, partial inhibition of the VLCFA deposition did not affect the Golgi dynamics.

**Figure 8:**
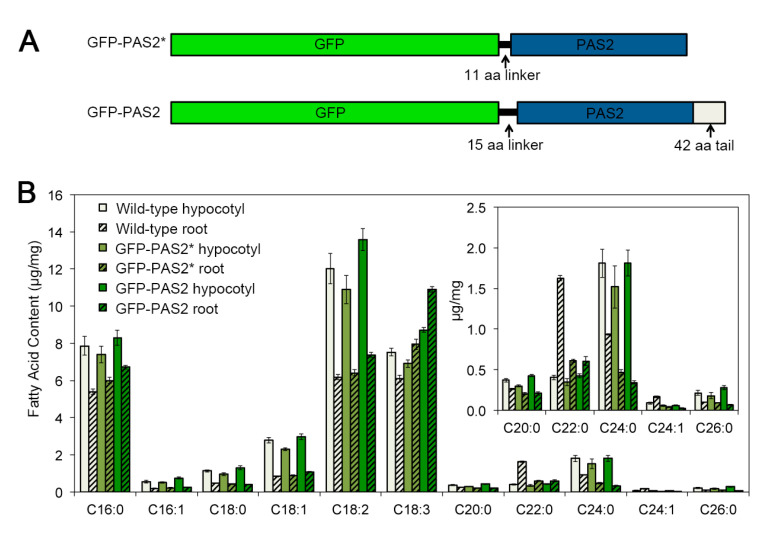
Comparison between the GFP-PAS2 transgenic line generated in this study (GFP-PAS2) and that generated by Bach et.al (GFP-PAS2*). (**A**). Schematic graphs showing two different GFP-fusion constructs of PAS2. (**B**). FAMES analysis of 4-day-old etiolated hypocotyls and the roots of 15-day-old light-grown seedlings. Both GFP-PAS2 and GFP-PAS2* seedlings have comparable VLCFA deposition to wild-type seedlings in etiolated hypocotyls, while showed decreased VLCFA (C22:0, C24:0, C24:1) content in roots. Error bars represent SD.

However, our observation of PAS2 localization is inconsistent with Morineau et al., who generated a fluorescence fusion protein of PAS2 (will be denoted as GFP-PAS2* for the rest of the paper for clarification), which localized exclusively in ER [48]. The fluorescence fusion construct GFP-PAS2* was similar to our construct in that a 1.5–2.0 kb native promoter drove the expression of PAS2 cDNA fused with GFP. However, the linker sequence between GFP and PAS2 in our GFP-PAS2 construct was 4 amino acids (aa) longer than the GFP-PAS2* construct (15 aa compared to 11 aa); secondly, in our GFP-PAS2, the stop codon of the *PAS2* gene was removed, which led to a 42-aa tail (Figure 8A). These differences might result in different subcellular localizations of PAS2.

To clarify which construct reflects the bona fide subcellular localization of PAS2 in the *Arabidopsis* hypocotyl cells, we conducted further investigations on the transgenic lines of these two constructs. We firstly checked the phenotypic complementation of these transgenic lines, and found that both constructs partially complemented the *pas2-1* mutants’ elongation defect in both etiolated hypocotyls and roots. The two transgenic lines are comparable in the length of hypocotyls and roots, and were both mildly shorter than wild type seedlings (Figure 2A,B,D,E). Interestingly, although the crystallized cellulose content in the hypocotyls of both lines were comparable to that of wild type seedlings, cellulose content in the roots were both reduced (Figure 2C,F). We also performed a FAMEs assay to evaluate the fatty acid composition in both transgenic lines. Intriguingly, both transgenic lines showed normal fatty acid content in the hypocotyls (Figure 8B), but neither of the two lines fully rescued the VLCFA content in roots. They both showed reductions in the long chain fatty acid pools (C20:0, C22:0, C24:0, C24:1 and C26:1), as well as accumulations of some of the short chain fatty acid (C16:0, C18:2 and C18:3) (Figure 8B). These data further support that VLCFA content in etiolated hypocotyls and light-grown seedlings roots are differently regulated. Changes of the VLCFA pool in hypocotyls and roots are not always synchronized. Since both GFP-PAS2 and GFP-PAS2* complemented the cellulose synthesis defect in the etiolated hypocotyls, it is difficult to judge which GFP-fusion protein represents the authentic subcellular localization in hypocotyls. 

## 3. Discussion

Several lines of evidence suggest PAS2 is a multifunctional protein. PAS2 shares sequence homology with multiple proteins such as protein tyrosine phosphatase-like (PTPL) and 3-hydroxy-acyl-CoA dehydratase [33,34,38]. PAS2 interacts with multiple proteins including cyclin dependent kinase A [35] and CESA (this study). Consistent with its multifunctional activity, *pas2-1* showed pleotropic defect including altered embryo, leaf and root development [30]; altered cell division and cell elongation [33,45]; loss of cell adhesion; altered responses to cytokinin [39]; and disorganized cell proliferation [34]. Some of these developmental defects are associated with PAS2′s role in VLCFA production. It has been shown recently that VLCFA is critical for the regulation of plant development, cell division and cytokinin production [38,45,49]. In this study, we showed that *pas2* mutation led to defects in cellulose synthesis in primary cell walls characterized by reduced cell elongation in dark-grown hypocotyls, reduced cellulose content, reduced motility of CSCs at the plasma membrane and reduced delivery rate of CSCs (Figure 2 and Figure 3). Given the well-characterized defect in the distribution of VLCFA pools in *pas2* mutants, it is possible that the abnormal dynamics of CSCs in *pas2* mutants is due to disrupted plasma membrane dynamics and/or compromised trafficking pathways. CSCs are transmembrane complexes that are only active at the plasma membrane. Therefore, their functionality relies on the integrity of plasma membranes. VLCFA is primarily present in sphingolipids that serve as structural components in the lipid bilayers in endomembranes and plasma membrane in plants. Therefore, membrane dynamics in plant cells can be regulated by the deposition of very long acyl chain sphingolipid [50]. Alternatively, the defect in the distribution of VLCFA pools in *pas2* affect the secretory pathway of CSC trafficking from Golgi to the plasma membrane. Consistent with this idea, the VLCFA-containing sphingolipids are required for the secretory targeting of the auxin transporters AUX1 and PIN1 to the plasma membrane [51]. 

VLCFA has also been proposed to have a signaling role in response to biotic and abiotic stresses [52]. When the content of VLCFA is below a threshold level, it may trigger signaling responses that lead to a reduced mobility of Golgi in *pas2-1*, thus affecting cellulose production and plant growth. Glycosyl-inositolphosphoryl-ceremides (GIPCs) represent the most abundant sphingolipids that are poorly characterized in plants. The GIPC backbone is synthesized in ER, and it is further modified in Golgi via glycosylation. A recent study showed that lesions in GIPC MANNOSYL-TRANSFERASE 1 (GMT1) had reduced GIPC glycosylation. Although Golgi-synthesized cell wall polysaccharides were unaffected, *gmt1* mutant had reduced cellulose content and stunted growth including dark-grown hypocotyls [53]. It is interesting to speculate that changes in composition and/or levels of VLCFA-containing lipid such as GIPC may affect assembly and/or modification of CSC in the Golgi. Overall, VLFCA dehydratase directly and/or indirectly regulates CSC and cellulose production.

Interestingly, genetic alteration of the activity of VLCFA dehydratase by introducing a point mutant form of PAS2 leading to different effects on cellulose synthesis in etiolated hypocotyls versus in light grown seedling roots (Figure 5). The same transgenic line caused more significant reduction in VLCFA pool in roots than in hypocotyls. These observations indicate that the activity of PAS2 is differentially regulated with tissue specificity. Accordingly, both cellulose production and cell elongation were inhibited in roots, while they remained unaffected in hypocotyls. This indicates that a minimum level of VLCFA in hypocotyl is sufficient for some of its cellular function—e.g., cellulose production. However, our data also showed that in the etiolated hypocotyls, pharmacological inhibition of VLCFA elongation by application of flufenacet, which mimicked the *pas2-1* mutant and consistent with the reduced VLCFA content similar to that of *pas2-1* mutants, did not influence the plasma membrane CSCs dynamics, nor did it affect the crystalline cellulose content (Figure 6). We hypothesize that, in addition to its role VLCFA synthesis, PAS2 may have a novel function in regulation of CSC. 

The subcellular localization of PAS2 has been examined by several groups. Utilizing fluorescent protein tagging of PAS2, the subcellular distribution of PAS2 was examined both in BY-2 cell lines and in *Arabidopsis* root cells [35,38]. GFP-PAS2 localized in the nucleus or the perinuclear region in two different cell types [35]. However, the pattern of transgene expression driven by CaMV35S promoter is prone to non-specific regulation and might not truly reflect its native localization. In a follow-up study, a native promoter-driven GFP-PAS2 was used to detect PAS2 localization in tobacco epidermal cells, where they extensively localized in ER [38]. In our study, a functional native promoter-driven GFP-PAS2 was transformed into the *Arabidopsis pas2-1* mutants. We found that in addition to its localization in ER, which is consistent with what was observed in tobacco [38], GFP-PAS2 also resides in the Golgi (Figure 7). The inconsistency of the GFP-PAS2 subcellular localization could be due to structural differences between these two fusion constructs. The GFP-PAS2 construct in this study contains a longer, more flexible linker sequence between GFP and the PAS2 protein; and it also harbors a peptide tail at the C terminal of PAS2 (Figure 8). Although these differences might potentially cause improper folding of either or both fusion proteins, we found that in the hypocotyls of both transgenic lines, the cell expansion, cellulose biosynthesis and VLCFA deposition were all rescued to the level of wild type, suggesting that both versions of fusion proteins could function normally in dark-grown *Arabidopsis* hypocotyls. Further proteomic studies are necessary to investigate the localization of native PAS2 protein in plant cells, and more importantly, whether Golgi represents the bona fide localization of the PAS2 in the *Arabidopsis* cells.

In higher plants, CSCs have not been detected in ER. Rosette CSC structure has been observed at the trans-side of Golgi and in Golgi-derived vesicles in electron micrographs [25], in the middle and the trans-side of Golgi in confocal microscopy [3,10,11,12]. Thus, it has been suggested that Golgi apparatus are where assembly and modifications of CSCs take place. The mechanism of CSC assembly in Golgi remains a mystery. The direct interaction between PAS2 and CESA by yeast two-hybrid assay and in vitro pull-down assays suggests that PAS2 might play a role by directly contacting with CESAs, in CSC assembly, modification or transporting CSC through the Golgi. Consistent with this hypothesis, the secretion rate of CSCs was drastically reduced in *pas2-1* mutants (Figure 3), which indicates either a defect in the efficiency of CSC assembly or PAS2 might play a direct role in Golgi-associated delivery of CSCs to the plasma membrane. However, it is intriguing that in the GFP-PAS2 line generated by Bach et al., where no apparent PAS2 signal could be detected in Golgi, cellulose biosynthesis is not affected. This argues against the role of PAS2 in Golgi-related function. The localization of GFP-PAS2 in the Golgi revealed by this study is surprising and unconventional as PAS2′s established function resides in ER [38]. It was recently shown that transport of CESA from ER to Golgi is specifically regulated [54]. Given that PAS2 resides in both ER and Golgi and it physically interacts with CESA, it is reasonable to speculate that PAS2 has a novel regulatory role in ER and/or Golgi and it consequentially influences the efficiency of CSC delivery to the plasma membrane. The comparison of effects between *pas2* and flufenacet treated etiolated hypocotyls on CSC behaviors and cellulose content support a VLCFA-independent role of PAS2 in the regulation of CSC.

In summary, PAS2 represents a novel CESA interacting protein that influences the CSC trafficking from the Golgi to plasma membrane. In addition to its previously reported localization to the ER, PAS2 also localized to Golgi, indicating a CSC-related function independent of its role in VLCFA synthesis. 

## 4. Methods

### 4.1. Plant Material and Growth Conditions

*Arabidopsis thaliana* seeds were sterilized using 30% bleach, stratified at 4 °C for 3 days, and then plated on Murashige and Skoog (MS) plates (1/2 × MS salts, 0.8% agar, 0.05% MES, pH 5.7). For etiolated seedlings, plates were put in darkness at 22 °C and grown for specified number of days. For soil-grown plants, seedlings were germinated and grown under light on MS plates containing 1% sucrose for several days and then transferred to pots, grown in a growth chamber (Percival, Perry, GA, USA) at 22 °C under a 16-h-light and 8-h-dark cycle.

### 4.2. Transgenic Lines

The *pas2-1* mutants are EMS alleles in Col-0 background. To generate the P_PAS2_::GFP-PAS2 (GFP-PAS2) transgenic line, a DNA fragment containing 1.5-kb *PAS2* promoter and the full-length cDNA clone of *PAS2* were amplified using primers in Table S1 online and cloned into the PCR8/GW/TOPO vector (Thermo Fisher Scientific, Waltham, MA, USA). After sequencing, both fragments were cloned to the *pH7WGF2* vector [55]. The verified construct was introduced into *pas2-1* using *Agrobacterium tumefaciens*-mediated transformation [56]. A GFP-PAS2-expressing *Arabidopsis thaliana* transgenic line was crossed with one expressing mCherry-SYP32, -Got1p, -VTI12, -RabA1g or -RabG3f [46] to generate dual labeled transgenic lines, respectively. The point mutations were generated directed on the GFP-PAS2 construct using a QuikChange^®^ Site-Directed Mutagenesis Kit (Agilent, Santa Clara, CA, USA). The mutated constructs were transformed into *pas2-1* heterozygous background. The T1 generation was screened for *pas2-1* homozygous plants. A GFP-CESA3-expressing *Arabidopsis thaliana* transgenic line [3] was crossed with *pas2-1* heterozygous plants. F2 seedlings homozygous for *pas2-1* by PCR genotyping were used for confocal imaging and other analysis. 

### 4.3. Split-Ubiquitin Yeast Two-Hybrid Assay

PAS2 constructs were cloned into *NubGX33* using primers in Table S1 online. CESA3 and CESA6 were constructed into *CubPLV* as described previously [57]. *Saccharomyces cerevisiae* strain *THY. AP4* was used to cotransform *Nub* and *Cub* constructs as described previously [58]. *NubWT* was used as a positive control. *NubGX33* was used as a negative control. Cotransformants were grown at 30 °C for up to 3 d and were selected on synthetic medium lacking Trptophan (Trp) and Leucine (Leu). For growth assays, cells were grown on synthetic medium lacking Trp, Leu and Histidine (His). β-Galactosidase activity was determined by filter lift assay. 

### 4.4. Cellulose Content Measurement

Homozygous *pas2-1* seeds cannot be maintained because *pas2-1* mutants are seedling lethal. Therefore, F2 seeds generated from the *pas2-1/+* heterozygous plants were plated on MS plates and grown in the dark for 4 days or under light for 15 days (light grown seedlings were kept in dark for 24 h for de-starching before collecting roots). *pas2-1* homozygous seedlings with severe mutant phenotype were selected for cellulose content measurement. For all other genetic backgrounds, etiolated 4-d-old homozygous seedlings or those under light for 15 days were collected from MS plates (light grown seedlings were kept in dark for 24 h for de-starching before collecting roots). Crystallized cellulose was measured using the Updegraff method [41]. Data were collected from five technical replicates for each tissue sample. 

### 4.5. Live Cell Imaging

Seedlings were grown vertically on MS plates in dark for 2.5 days. Images were taken from the epidermal cells in the regions about 3–7 cells below the apical hook. Imaging was performed on a Yokogawa CSUX1 spinning-disk system featuring a DMI6000 Leica motorized microscope, a Photometrics QuantEM: 512SC CCD camera and a Leica 100 × 1.4 numerical aperture oil objective (W.NUHSBAUM, McHenry, IL, USA). An ATOF laser with three laser lines (440/491/561/nm) was used to enable fast switching between different excitations. Band-pass filters (520/50 nm for GFP, 620/60 nm for RFP) were used for emission filtering. Image analysis was performed using Metamorph (Molecular Devices, San Jose, CA, USA), ImageJ (version 1.36b; http://rsbweb.nih.gov/ij/) and Imaris (Bitplane, Zurich, Switzerland) software. 

For CESA particle dynamics analyses, 5-min time series movies with 5 s interval were obtained from several cells in each genotype. The average intensity projection of 5-min time series was used to visualize the tracks underlined by CESA particles in ImageJ. Velocities of CESA particles were measured using kymographs. For the colocalization analyses, single-frame images from both channels (520/50 nm for GFP and 620/60 nm for RFP) were taken simultaneously. Images obtained were then processed using ImageJ for the detection of signals overlapped with one another. For the organelle particle motility analyses, time series movies of 1 s interval and of 21 s duration were obtained. Organelle particle tracks were underlined using the TrackMate plugin in Fiji (http://fiji.sc/TrackMate). 

For CESA particle density analyses, a single optical section image was taken at the plasma membrane focal plane. In imageJ, a region of interest (ROI) avoiding Golgi signal-masked area was selected using the “Freehand selections” tool. The area of ROI was determined by the “Measure” function, and the CESA particles within the ROI were detected using the “find maxima” tool.

For the colocalization analyses, the overlap of manually selected donut-shaped particles within a ROI was analyzed as a percentage of one particle population that overlapped with the other and vice versa.

### 4.6. Drug Treatments

Flufenacet (FA) was dissolved in Dimethylformamide (DMF) to create a 10 mM stock solution. For both cellulose content measurement and live-cell imaging, *Arabidopsis* Col-0 seeds were plated on MS plates containing 75 nM Flufenacet. For mock treatment, seedlings were grown on plates containing appropriately diluted DMF solution.

### 4.7. Lipid Analysis

Three replicates (2 mg) of each dry sample were used for quantification of the fatty acids methyl ester (FAMEs) by GC-MS as described by [59]. 

### 4.8. Protein Purification

The *PAS2* full-length coding sequence and three *PAS2* fragments were cloned into YG201, which contains a His tag, and expressed in BL21 *Escherichia coli*. Protein expression was induced with 1 mM isopropyl β-d-1-thiogalactopyranoside at 15 °C for 20 h. Protein purification was performed as described previously [27]. The coding sequences of the central domains of CESA1 and CESA3 were cloned into the pGEX-KG vector in frame with a GST tag and expressed in *E. coli*. Protein was induced with 1mM isopropyl β-d-1 thiogalactopyranoside at 15 °C for 4 h. Protein purification was performed as described previously [27].

### 4.9. In Vitro Pull-Down Assay

Resin-bound GST-CESA1 and GST-CESA3 proteins were washed three times in interaction buffer (20 mM HEPES, pH 7.4, 1 mM EDTA, 5 mM MgCl2, 1 mM dithiothreitol and 0.1% Triton X-100) for equilibration. Aliquots of approximately 10 μg of equilibrated GST-CESA proteins were incubated with approximately 10 μg of soluble His-PAS2/PAS2^1-80^/PAS2^81-140^/PAS2^141-221^ proteins in a total volume of 0.5 mL of interaction buffer for 2 h at 4 °C on a rocker. The resin was then washed eight times with interaction buffer, and resuspended in SDS loading buffer, boiled for 5 min and subjected to SDS-PAGE and Western blotting for analysis. For Western blots, His-PAS2/PAS2^1-80^/PAS2^81-140^/PAS2^141-221^ proteins were detected on film using a horseradish peroxidase-conjugated His antibody and SuperSignal West Femto substrate (Thermo Fisher Scientific, Waltham, MA, USA).

### 4.10. Accession Numbers

Sequence data from this article can be found in the *Arabidopsis* Genome Initiative or GenBank/EMBL databases under the following accession numbers: AT5G10480 (PAS2), AT4G32410 (CESA1), At5g05170 (CESA3).

## Supplementary Materials

The following are available online at https://www.mdpi.com/2223-7747/9/11/1599/s1, Figure S1: The *pas2-1* mutant does not affect the dynamics of TGN/EE or endosomal vesicles. Figure S2: Alignment of Arabidopsis PAS2 and its homologs in other species. Figure S3: *PAS2^Y156A^* did not affect the Golgi localization in etiolated hypocotyls but did affect VLCFA production in roots. Figure S4: PAS2 does not localize in trans-Golgi network/early endosome, late endosome/vacuole, recycling endosome or smaCCs/MASCs. Table S1: Primers used in this study. Video S1: At the plasma membrane of *pas2-1* hypocotyl cells, GFP-CESA3 particles moves along mCherry-TUA5 labeled cortical microtubules, but with lower velocity. Video S2: The delivery of CSC particles is delayed in the *pas2-1* mutants. Video S3: *pas2-1* has defects in Golgi dynamics. Video S4: GFP-PAS2 particles exist in both ER and Golgi-like structures. Video S5: GFP-PAS2 particles associate with mCherry-CESA3 particles in Golgi. Video S6: PAS2 does not localize to smaCCs/MASCs. Video S7: GFP-PAS2^Y156A^ has comparable Golgi dynamics as that of GFP-PAS2.
