# Supplementary material for: Disruption of Very-Long-Chain-Fatty Acid Synthesis Has an Impact on the Dynamics of Cellulose Synthase in Arabidopsis thaliana
Source: Plants (Basel). 2020 Nov 18;9(11):1599. doi: 10.3390/plants9111599 (PMC7698757; doi:10.3390/plants9111599)
Supplement: Supplementary file 1 [file plants-09-01599-s001.zip › Sup_Table1.docx]

| **Mutant genotyping** | **Forward primer** | **Reverse primer** |
| --- | --- | --- |
| *pas2-1* | TAAGCCATTTCCCTCAGACTC | CTTTCGCTGACCAAGCATGTACC |
| **Expression constructs** | **Forward primer** | **Reverse primer** |
| CESA1CD | GGATCCCAGTTTCCCAAATGGTAC | GTCGACTCAGACGATGGTGTTGAT |
| CESA3CD | GGATCCGATCAGTTTCCCAAGTGG | GTCGACTCAGATGGTGGTGTTCAC |
| PAS2 | GGGATCCATGGCGGGCTTTCTCTCCG | CGTCGACTTATTCCCTCTTGGATTTGGAGAG |
| PAS2^1-80^ | GGGATCCATGGCGGGCTTTCTCTCCG | CGTCGACTACTGTGGCAGAGTTGCAGAAACAGG |
| PAS2^81-140^ | GGGATCCATAGGTTCAAGGCTATTTCTCAC | CGTCGACTAGCTGTATCTGAGCCACAAGTGCC |
| PAS2^141-221^ | GGGATCCAGCTTTTTATTGCTATACCCTACCGG | CGTCGACTTATTCCCTCTTGGATTTGGAGAG |
| **Point mutagenesis** | **Forward primer** | **Reverse primer** |
| GFP-PAS2^Y156A^ | GCAGCTTTTTATTGCTAGCCCCTACCGGTATCACCAGC | GCTGGTGATACCGGTAGGGGCTAGCAATAAAAAGCTGC |
| GFP-PAS2^E163A^ | CCGGTATCACCAGCGCAGTTGGTCTTATCTACC | GGTAGATAAGACCAACTGCGCTGGTGATACCGG |
| **GFP-PAS2 construct** | **Forward primer** | **Reverse primer** |
| *PAS2 promoter* | CAAGCTTTAGACGTTCCTTTTCCTGT | GACTAGTGGAAAGTGAATACGCGAGA |
| *PAS2 CDS* | ATGGCGGGCTTTCTCTCCGTTGT | TTCCCTCTTGGATTTGGAGAGAGC |
| **Y2H construct** | **Forward primer** | **Reverse primer** |
| NubG-PAS2 | TATTCACTTTCCATGGCGGG | TTATTCCCTCTTGGATTTGGAGAG |
