# Supplementary figures and images for: Disruption of Very-Long-Chain-Fatty Acid Synthesis Has an Impact on the Dynamics of Cellulose Synthase in Arabidopsis thaliana
Source: Plants (Basel). 2020 Nov 18;9(11):1599. doi: 10.3390/plants9111599 (PMC7698757; doi:10.3390/plants9111599)

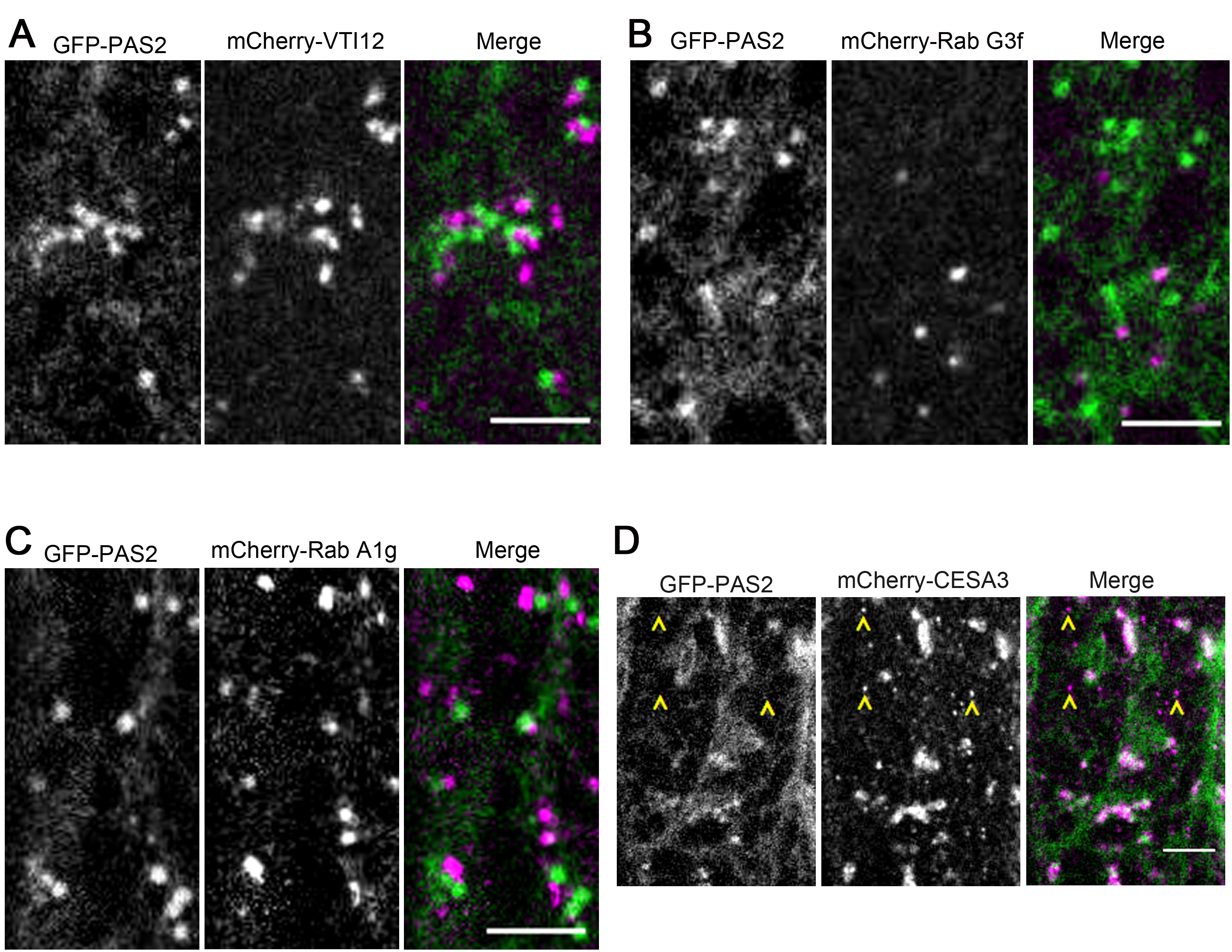

Supplement: Supplementary file 1 [file plants-09-01599-s001.zip › V16_sup_4.tif]

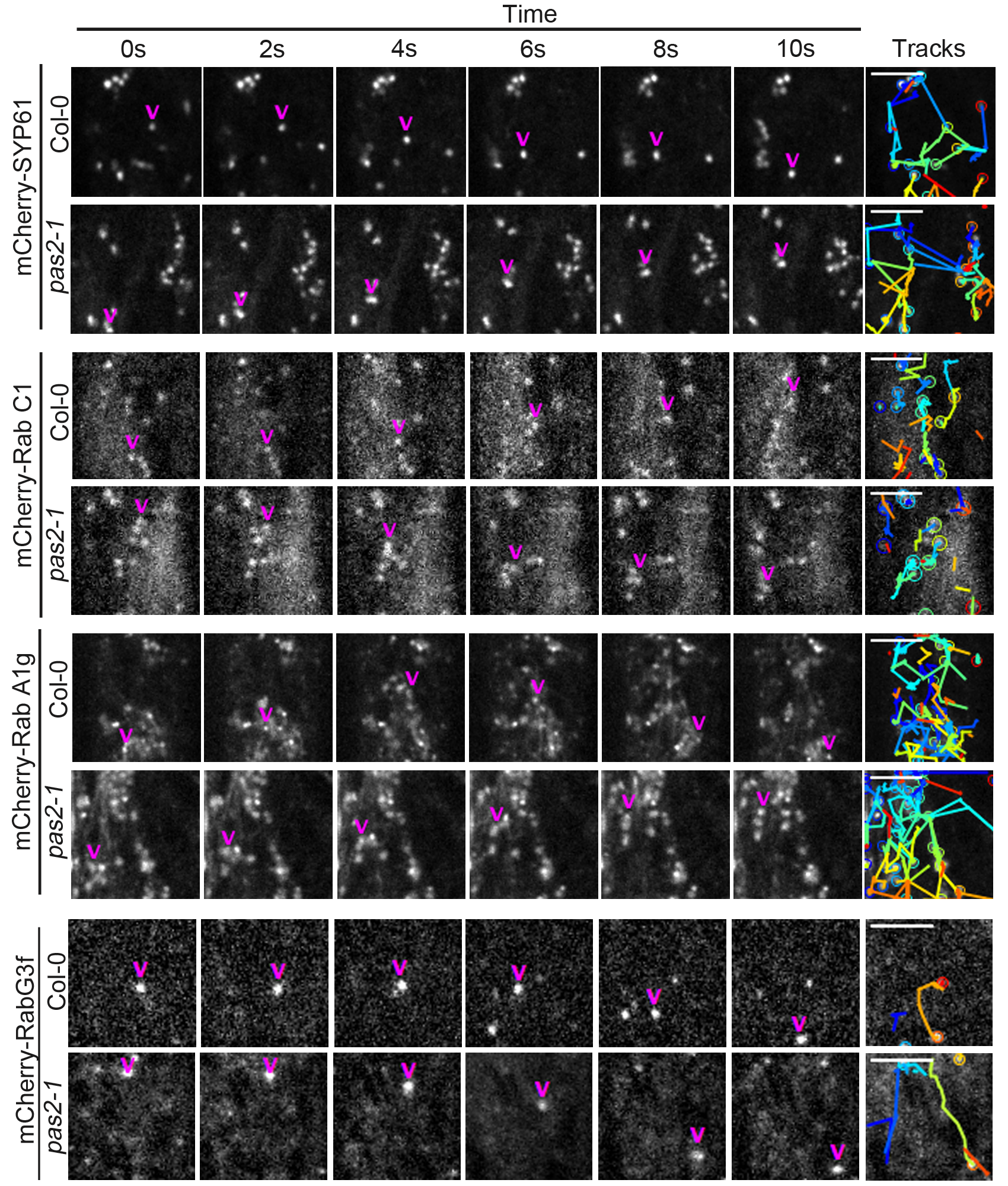

Supplement: Supplementary file 1 [file plants-09-01599-s001.zip › V16_sup_1.tif]

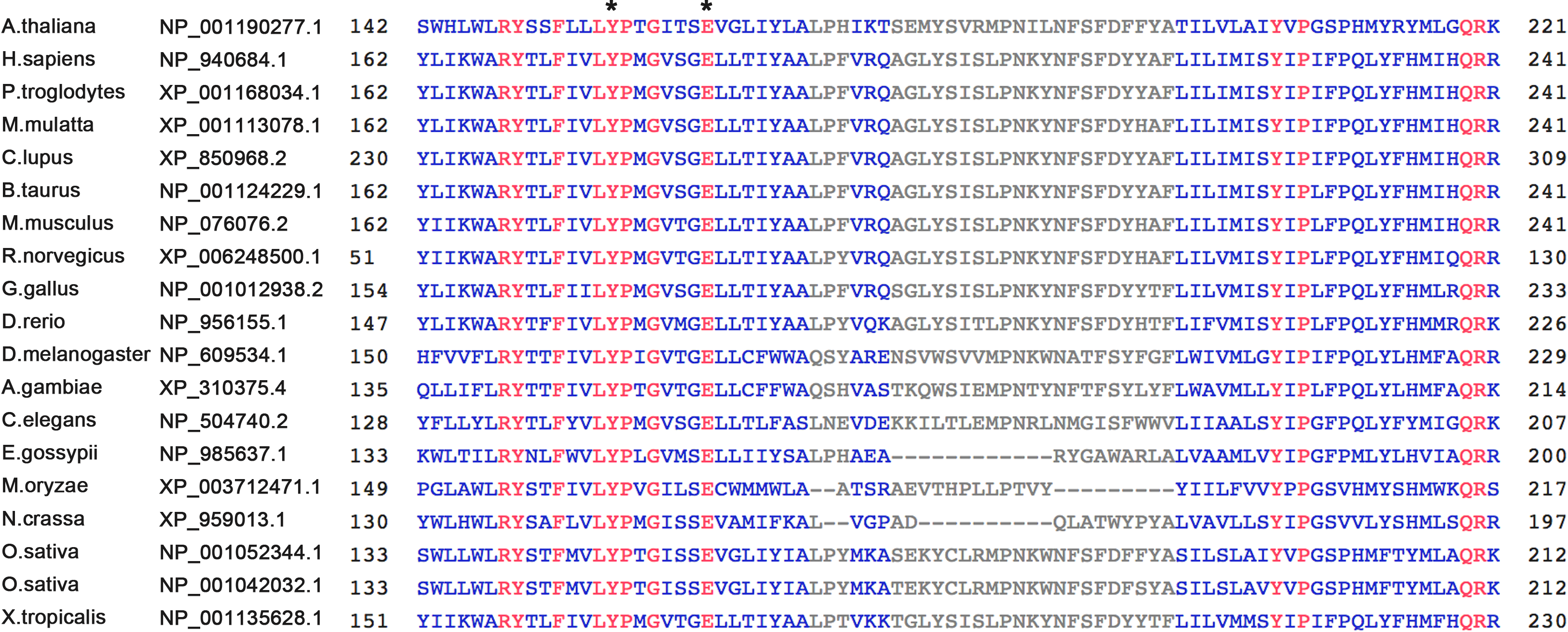

Supplement: Supplementary file 1 [file plants-09-01599-s001.zip › V16_Sup_2.tif]

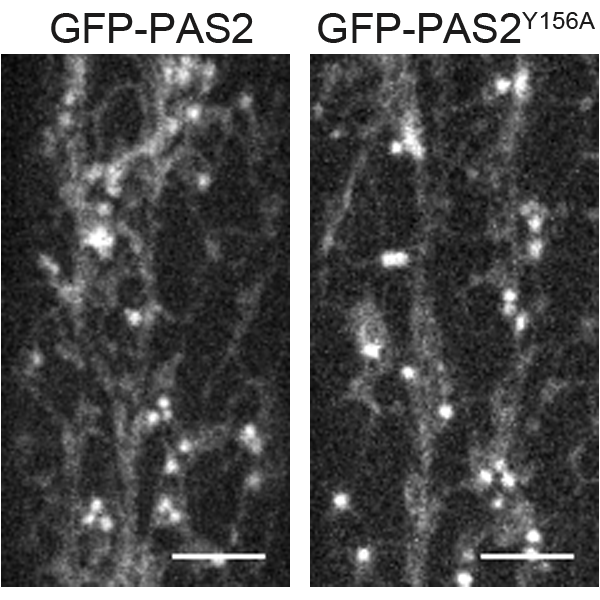

Supplement: Supplementary file 1 [file plants-09-01599-s001.zip › V16_Sup_3.tif]
